# Novel approach for treating challenging implant-borne maxillary dental rehabilitation cases of cleft lip and palate: a retrospective study

**DOI:** 10.1186/s40729-022-00401-x

**Published:** 2022-02-02

**Authors:** Björn Rahlf, Philippe Korn, Alexander-Nicolai Zeller, Simon Spalthoff, Philipp Jehn, Fritjof Lentge, Nils-Claudius Gellrich

**Affiliations:** Department of Oral and Maxillofacial Surgery, Hannover Medical School, Carl-Neuberg-Str. 1, 30625 Hannover, Germany

**Keywords:** Cleft lip and palate, IPS-preprosthetic®, Implant-borne dental rehabilitation, 3D-technology, Selective laser melting, CAD/CAM, Dental implants

## Abstract

**Purpose:**

Dental restoration in cleft lip and palate (CLP) patients is demanding and often results in bone loss and dental implant failure. Furthermore, unfavorable conditions of hard and soft tissues as well as skeletal deformities aggravate surgical and dental treatment. Therefore, this study was designed to assess the feasibility of using a new type of patient-specific implant (IPS-preprosthetic®) in CLP patients.

**Methods:**

Of the 63 patients who received a IPS-preprosthetic® implant in the Department of Oral and Maxillofacial Surgery at the Hannover Medical School, Germany, six patients were treated for a CLP deformity with significant soft and hard tissue impairment. Two patients were partially edentulous, whereas four patients were edentulous for the maxilla. All implants were inserted in a single-step outpatient surgery and were followed up for up to 40 months.

**Results:**

Within the observation period, no implant failed and no screw loosening or change in stability of the implant to recipient site occurred (mean number of screws: 21). This study demonstrates, for the first time, the efficient use of a one-piece multivector screw primarily retained a stable patient-specific implant for implant-borne prosthodontic rehabilitation of CLP patients with deformities and challenging initial situations.

**Conclusions:**

IPS-preprosthetic® implants offer a novel approach to implant dentistry treatment protocols, especially in difficult cases of unusual anatomy, even when previous conventional treatment fails.

## Background

Dental restoration in patients with a cleft lip and palate (CLP) remains challenging due to soft tissue and hard tissue problems resulting from congenital deformities and previous surgical treatment [1]. Furthermore, growth disturbances due to a class III skeletal angle malocclusion often occur in these patients, requiring orthodontic treatment [2]. The typical treatment protocol for CLP patients includes the finalization of primary surgical closure in early childhood [3]. However, due to an unfavorable tooth position, a loading or growth disturbance with malocclusion and an early or late loss of dentition up to an edentulous situation, especially in the maxilla, might follow. This is even worse in cases of a more or less fully dentated mandible, which negatively contributes to the eccentric type of occlusal loading [4].

Standard treatment protocols for reestablishing dentition typically consist of four steps. First, the compromised and deficient bone is replaced, typically with non-vascularized bone grafting from the iliac crest. In some severe cases, this is achieved using microvascular bone transfer from the iliac crest, fibula, or scapula. Second, conventional dental implants are placed into the osseointegrated bone grafts. Third, the dental implants are uncovered. Finally, in cases of insufficient keratinized gingiva around the implant shoulders, free mucosal grafting is performed.

The total treatment time from bone grafting to dental implant insertion up to the final prosthodontic solution is approximately 1 year [5]. Notably, previous clinical studies have found that the volume of augmented bone within the cleft area decreases over time leading to failure of the treatment mentioned in the above protocols [6]. Furthermore, in cases of peri-implantitis, unfavorable biomechanical loading with implant fracture or loss, bone loss, and negative crossfire caused by soft tissue complications such as remaining oronasal fistulas, either incomplete or complete loss of the formerly applied implant-borne dental restoration might occur [7]. Therefore, in these cases, bailout surgery must be considered.

We recently described a new technique based on digital prosthodontic backward planning with a primary and functionally stable one-piece implant manufactured using selective laser melting technology consisting of a titanium grade 4 framework mounted with four posts in case of an edentulous maxilla rigidly fixated in a multivector screw-retained technique [8, 9].

**Fig. 1:**
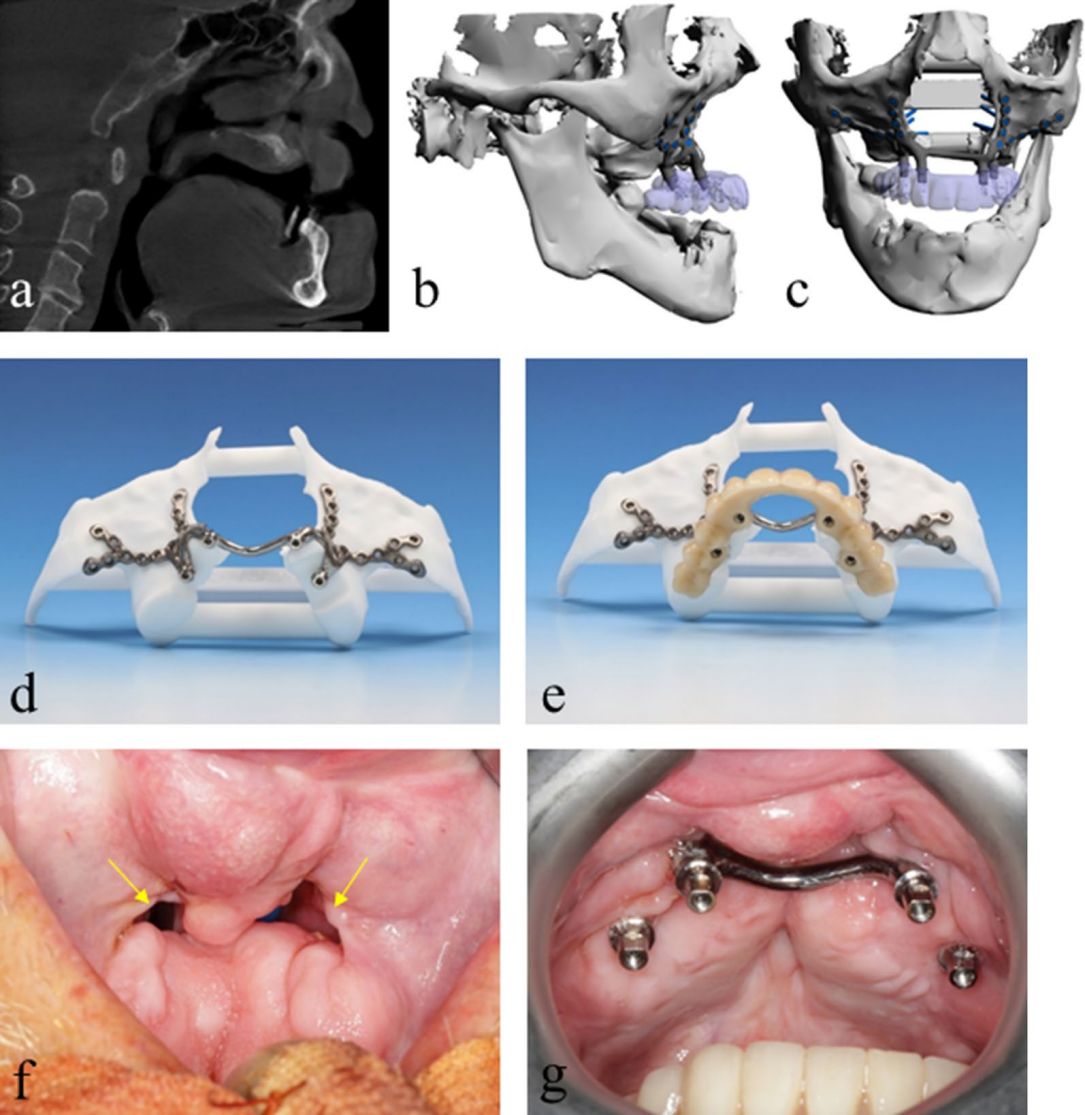
Radiologic and clinical presentation of a typical patient. Imaging results from a 68-year-old patient showing a compromised vertical and sagittal bony dimension in the remaining maxilla together with an extended anterior oronasal fistula. Sagittal screenshot of a cone beam CT showing the distinctive pseudo-class III relationship (**a**) and the backward planning of the posts considering the opposing arch of the mandible (**b**–**c**). An IPS-preprosthetic® implant mounted on a stereolithography model (**d**) with a temporary denture (**e**). A case of significant bony defect within an extended oronasal fistula in the anterior maxilla prior to implant-borne dental rehabilitation (**f**) and the following IPS-preprosthetic® placement where the connecting palatal bar bridges the anterior maxillary defect, which was digitally preplanned (**g**)

This implant, named IPS-preprosthetic® (KLS-Martin, Tuttlingen, Germany), was developed for oncological patients post-ablation and those with severely atrophic jaws. It has been demonstrated to be a feasible method for oral rehabilitation after ablative surgery in terms of clinical results and quality of life [10]. Due to the promising results in CLP patients, the methods for oral rehabilitation were adapted accordingly. Therefore, instead of pushing the traditional concept of grafting and implant insertion over the limits, we changed the protocol to use the IPS-preprosthetic®, especially in cases of difficult edentulous maxillas and in CLP patients, with the intention of reducing the total rehabilitation process towards a single-step outpatient-based surgical procedure with direct insertion of a temporary prosthesis (Fig. 1).

This method can also be used for patients who are not eligible for major procedures due to their general health status or due to a previously failed dental implant treatment.

## Methods

From March 2015 to July 2021, 63 patients were treated with a patient-specific implant (IPS-preprosthetic®, KLS-Martin, Tuttlingen, Germany) at the Hannover Medical School for either the maxilla or the mandible due to post-ablative, atrophic, posttraumatic, or congenital defects (Table 1).

**Table 1:** Distribution of the cases according to sex and indications

	Upper jaw		Lower jaw		Number
Sex	**♂**	♀	♂	♀	
Tumor	10	10	10	8	38
Atrophy	4	9	–	1	14
Cleft lip and palate	3	3	–	–	6
Trauma	2	–	–	–	2
Necrosis	1	1	–	1	3
Total	20	23	10	10	63

**Fig. 2:**
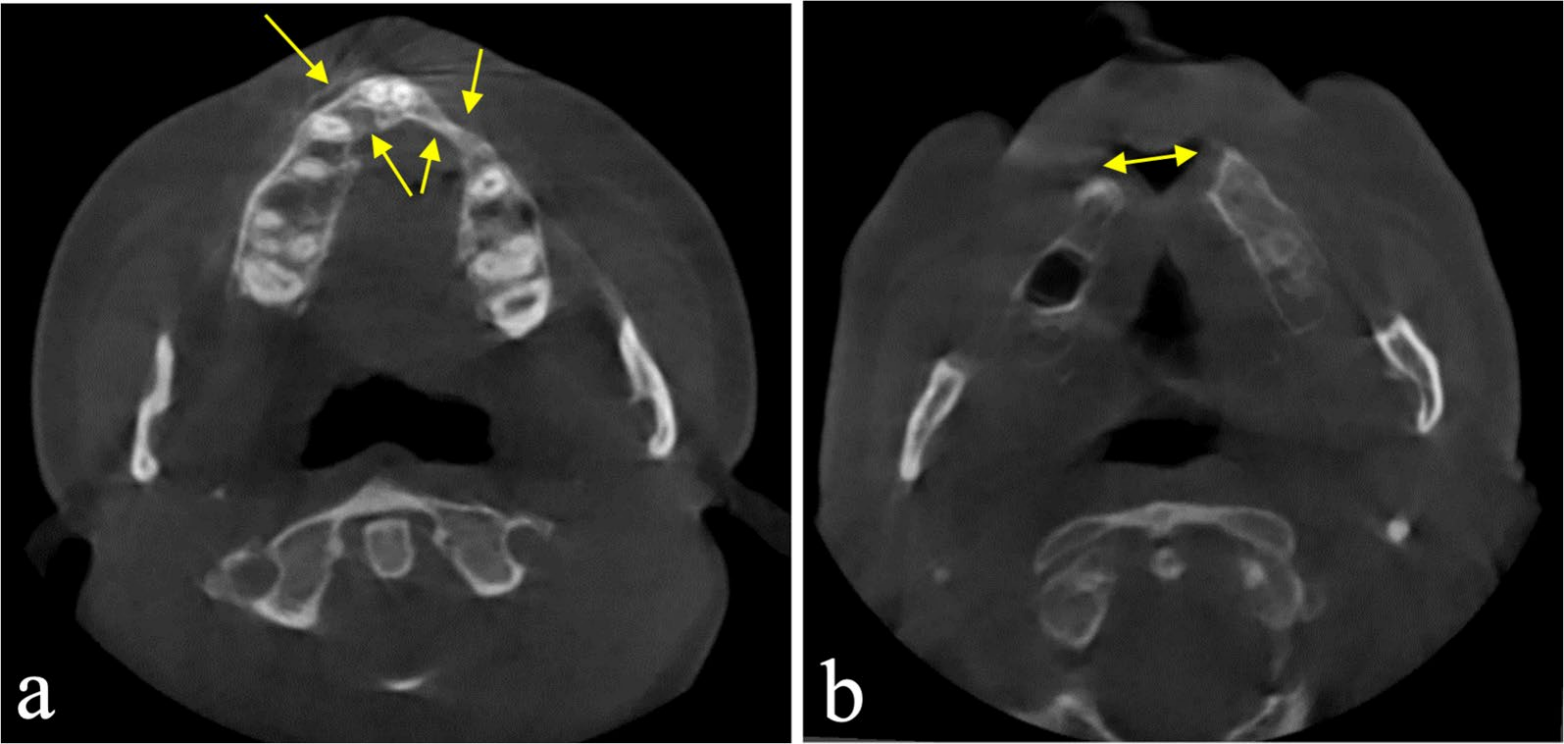
Typical radiologic findings in CLP patients. Cone-beam CT scan showing an axial slice with a formerly bone-grafted premaxilla displaying compromised horizontal bony dimensions for the left and right lateral incisor region (arrows) including the left canine in a partially edentulous 18-year-old female patient with van der Woude syndrome (**a**). Cone-beam CT scan from a patient following complete loss of the premaxilla during treatment for a CLP deformity elsewhere (**b**) (patient from Fig. 1)

Of the six patients treated with an IPS-preprosthetic® for the maxilla due to a CLP-associated deformity, two had maxillary partial edentulism (remaining number of teeth: 8 and 9) and four had total edentulism. Two examples are shown in Fig. 2.

In one case, the area of dental rehabilitation was addressed in only one quadrant, whereas in five cases, bilateral prosthodontic restoration was planned. Of these five bilateral cases, in one patient with partial edentulism, only the anterior teeth region was addressed. The patients ages ranged from 18 to 68 years, and sex was evenly distributed so that three men and three women were treated with this new type of patient-specific implant. Except for the youngest patient, all the other patients suffered from a previous complete loss of implant-borne restorations placed into the augmented bone. Therefore, the patients waved another approach for either conventional or implant-borne prosthodontic restoration as this would require them to undergo a major surgical intervention, including microvascular bone transfer. In all cases, a one-piece implant was used to combine 2–4 posts. The number of posts used was based on the number of remaining teeth in the upper jaw as well as the plan for prosthetic restoration in each patient. In two cases, the IPS-preprosthetic® was combined with conventional dental implants, which either had to be inserted or were already in place and thus included in the prosthodontic restoration. Typically, the soft tissues covering the IPS-preprosthetic® showed significant scarring following previous surgical interventions due to the cleft deformity. All cases showed severe Angle class III skeletal relationships; however, in one patient, a bimaxillary surgery had already compensated for the previously existing class III relationship. The other partially edentulous case showed significant compensation of the existing dental arch with protrusion of the incisors. The opposing teeth in the mandible were complete in the two youngest patients, and in the remaining four cases, removable dentures were fixed to either dental implants (two implants, *n* = 2; four implants, *n* = 1) or natural teeth (five teeth, *n* = 1). All patients were followed up at the outpatient clinic. In each of these six patients, prosthodontic digital backward planning with post position was feasible and could be performed at the level of outpatient-based one-step implant insertion and screw-retained fixation. The follow-up period ranged from 6 to 40 months (Table 2).

**Table 2:** Distribution of the CLP patients undergoing IPS-preprosthetic® treatment

Patient number	1	2	3	4	5	6
Sex	Female	Male	Female	Male	Female	Male
Age at surgery	18	64	66	33	56	68
Number of teeth remaining in the upper jaw	9	0	0	8	0	0
Extension of IPS-preprosthetic® posts uni- (u)/bilateral (b)	b	b	b	u	b	b
Opposing teeth in the lower jaw	Fully dentated	Removable dentures on 4 dental implants	Removable dentures on 5 teeth	Fully dentated	Removable dentures on 2 dental implants	Removable dentures on 2 dental implants
Angle class III relation	+	+	+	− Previous bimaxillary surgery	+	+
Oro-nasal fistula	−	+	+	+	+	+
Previously failed dental implant reconstruction	−	+	+	+	+	−
Previously failed/insufficient bone augmentation	+	+	+	+	−	−
Duration of surgery (min)	168	186	107	159	114	141
Trimming of the alveolar crest with CAD resection guide	−	−	+	−	−	+
Number of posts	3	4	4	2	4	4
Number of screws	23	20	20	16	23	21
Length of screws (mm)	5–11	5–13	5–11	5–9	7–11	7–9
Bichat fat pad	−	+	+	+	+	+
Framework-exposure around posts	2/3	3/4	1/4	0/2	0/4	0/4
Framework-exposure distant from posts	−	−	−	−	−	−
Loss of implant	−	−	−	−	−	−
Temporary dentures	−	−	−	−	−	+
Definitive dentures	+	+	+	+	+	−
Observation period (months)	40	22	16	15	10	6

*CLP* cleft lip and palate; *CAD* computer-aided design

All patients provided written informed consent for research participation as well for the publication of clinical data and pictures. Ethical approval was obtained from the institutional ethics committee of the Hannover Medical School (reference number: 8552_BO_K_2019).

## Results

**Fig. 3:**
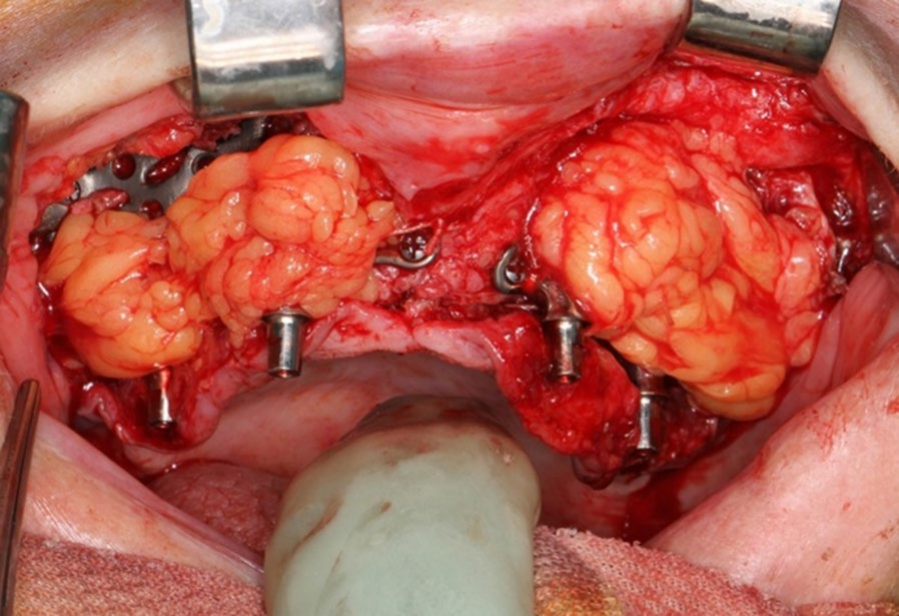
Soft tissue covering of the IPS-preprosthetic® implant. A Bichat´s fat pad flap is used for covering the buccal framework as a submucosal second layer

In all six cases, the healing process was uneventful despite the unfavorable soft tissue quality, including persisting oronasal fistulas in five cases (Fig. 1f). Notably, primary loading was applicable in all cases. We applied our method in combination with conventional dental implants in two cases, either simultaneously or consecutively, in the maxilla. In five cases of lateral maxilla construction, the implant posts and framework were shielded from the buccal side using a pedicled Bichat fat pad flap (Fig. 3).

The latter served as an efficient method to cover the metallic framework in a double-layer technique together with mucosal covering. In the four edentulous patients, mucoperiosteal advancement flaps were created with a minimum of 10 mm to allow for a better mucosal reconstruction around the implant posts and to allow for better separation of the anatomical units from the vestibule to the lip and cheek.

**Fig. 4:**
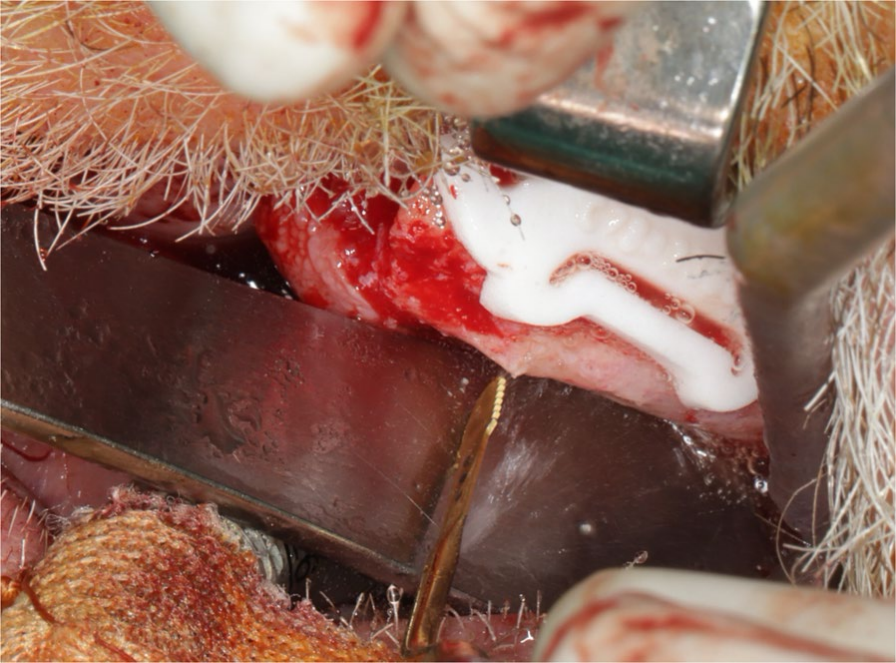
Leveling and trimming of the alveolar crest. Intraoperative view showing the serrated tip of the piezosurgery device (Mectron, Carasco, Italy) and the resection guide for trimming the alveolar crest prior to IPS-preprosthetic® placement

The duration of outpatient surgery ranged from 107 to 186 min. All surgeries were performed under general anesthesia. During surgery, either 10 million units of penicillin or 600 mg of clindamycin were intravenously administered. In the two edentulous patients, the irregular alveolar crest was trimmed using piezosurgery guided by a computer-aided design/computer-aided manufacturing (CAD/CAM) resection template made from autoclavable polyamide (Fig. 4).

Accordingly, the achieved technical form of the alveolar crest was already integrated into the design of the individual IPS-preprosthetic® that was simultaneously inserted during the same surgery. In all patients, the extension of the framework towards the transition zone between the malar bone and zygomatic arch included dissection of the anterior insertion of the masseter muscle. Furthermore, in all patients, primary rigid fixation was achieved by the application of 16–23 1.5-mm-diameter mini-screws (MaxDrive ® KLS Martin Group, Tuttlingen, Germany) ranging in length from 5 to 13 mm, instead of 1.8 mm diameter emergency screws. Notably, 16 screws were used in one patient who needed an IPS-preprosthetic®, whereas 20 or more screws were used in all other cases of bilateral maxillary reconstruction. Primary postoperative loading was unrestricted in all cases over the entire observation period. However, all of the patients had to gain confidence over time in loading the new implant because they had all had bad experiences due to previously insufficient conventional vs. implant-borne prosthodontic restorations.

**Fig. 5:**
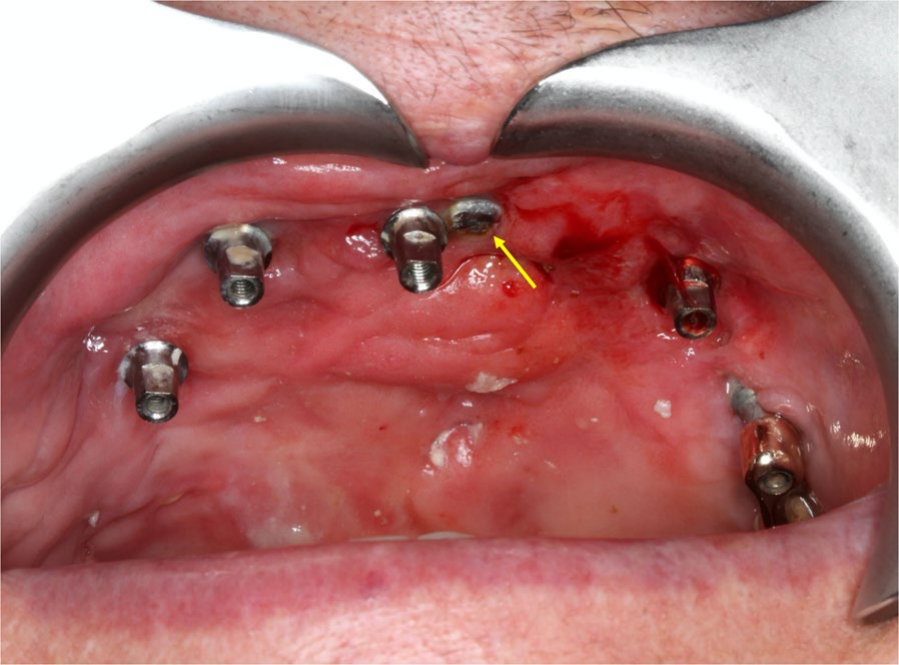
Partial exposure of the framework. The framework exposure partially (arrow) beside in six out of 21 posts, without any clinical problems apart from mucositis

Although partial exposure of the framework occurred around the transition zone in six out of 21 posts, it did not result in any clinical problems apart from mucositis (Fig. 5). Notably, in three patients, no framework exposure occurred.

**Fig. 6:**
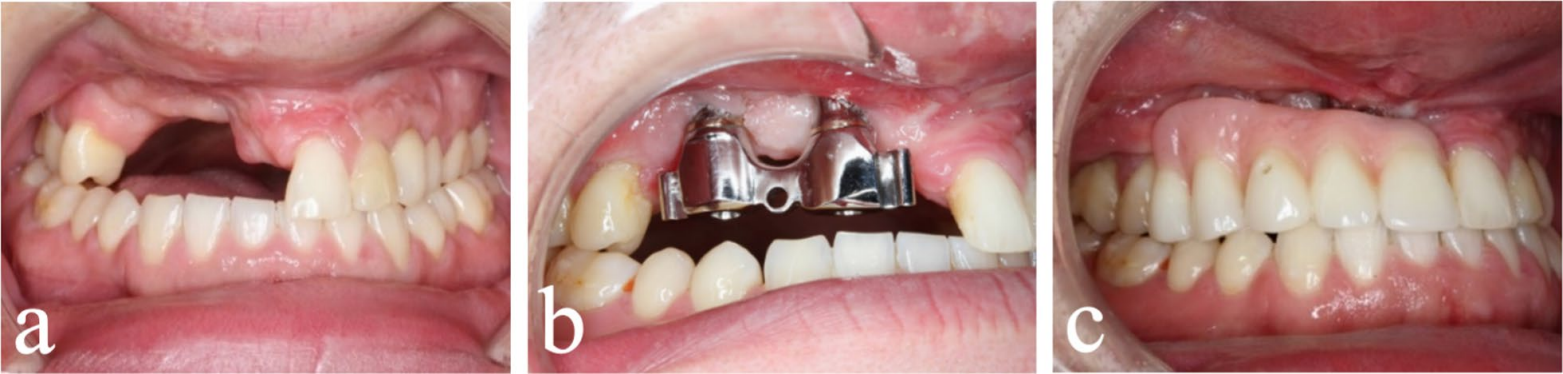
Treatment path to final prosthodontic restoration. Clinical images showing failure of a bone augmentation and the compensated dental arches and the compromised hard and soft tissues (**a**). Final prosthodontic restoration using a bar retained removable partial denture (**b**–**c**)

None of the inserted IPS-preprosthetic® implants were lost, and no screws loosened or needed to be removed within the observation period. In total, five patients underwent final prosthodontic restoration during the postoperative follow-up (Fig. 6).

One patient has not yet received the final prosthesis because of general health problems, including suffering a stroke.

## Discussion

Currently, any implant-borne dental restoration must be prosthodontically driven backward planned [11], especially in CLP patients [12]. However, the negative impact of an unfavorable skeletal relationship, such as Angle class III due to a growth deficiency in the maxilla, as well as the conditions of the patients soft and hard tissues, must be considered during the planning process.

Importantly, the status of the remaining dentition, as well as previous surgeries, scars, or tissue loss has to be taken into account, especially in older CLP patients. Furthermore, a retruded edentulous maxilla with or without oronasal fistula is often observed in patients over 50 years of age, with the opposing mandible often showing either full dentition or relatively good and biomechanically stable prosthodontic restorations. However, the more the imbalance between a strong mandible and a weak maxilla is stressed, the more likely it becomes that conventional prosthodontic approaches will be ineffective. In these cases, implant-borne prosthodontic restorations provide superior results [13]. Additionally, the use of conventional dental implants is limited by their dimensional needs and correct anatomical positioning [14]. Nevertheless, both approaches are strongly dependent on the required dimensions and quality of the underlying bone and adjacent soft tissues. Given that tissue quality is often compromised by previous surgical interventions, including bone grafting, soft tissue flaps, and scarring, prosthodontic restorations in the maxillary region of CLP patients, are challenging [15, 16]. Therefore, conventional implant-borne prosthodontic treatment approaches to fail over time in edentulous CLP patients. Ultimately, their prosthodontic approaches fail, which is the driving force behind our CLP patients seeking alternative treatment options.

Originally, the idea of IPS-preprosthetic® was born out of the demand for treating oral cancer patients with extended jaw defects. These defects were either due to ablation itself or failure of previous bone reconstruction approaches, where the patients refused to have another microvascular bone graft. However, patients after trauma or with extreme atrophy were also treated using this new method. So far we have been able to reconstruct all defects using this new method. Anchoring can also be guaranteed in difficult cases by using multivector and distant fixation. Historically, Hammer and Rohner were the first to develop a protocol for the osseointegration of dental implants in ectopic engineered fibular bone together with prelamination of the peri-implant region at the fibular site following a prosthodontically driven backward plan [17, 18]. Briefly, after osseointegration, the microvascular fibular bone was grafted into the maxillary defect, and the analogue prosthodontic backward plan was finalized by mounting the prosthesis onto the contoured microvascular fibular bone graft simultaneously at the time of transplantation and microvascular anastomoses. At that time, it was the most advanced and complex protocol allowing for adequate biological reconstruction.

In contrast to guided surgery protocols, where a technology-sensitive interface is required for accurate implementation of the plan within the clinical situation, the approach presented in this paper allows for a one-fits-all patient-specific implant that includes all of the information on parallel posts positions independent of the amount of remaining bone in the maxilla. Importantly, all other approaches depend on predetermining the required amount of bone in the prosthodontically defined post-position area.

Through milling processes and laser melting technology, it is possible to manufacture nearly any type of inner or outer implant design. This offers unprecedented flexibility in terms of the design features that can be utilized, which were not obvious or had not been considered before. For example, designs with flanges towards the piriform aperture that can be used as positioning aids, or that catch the transition zone between the malar bone and zygomatic arch, or designs with sloped ends of the basic framework. Furthermore, the thickness of the framework can be customized, and it is possible to use select from three different screw types, i.e., 1.5-mm non-locking, 2.0-mm locking, and 2.0-mm non-locking (KLS-Martin, Tuttlingen, Germany). Similar to any conventional dental implant treatment protocol, primary stability is fully provided at the end of surgery, where in the case of patients with an edentulous upper jaw, a total of 84 screws were used in four patients (ranging from 20 to 23 screws). Importantly, this multivector distant anchorage fixation protocol allows for rigid fixation without biomechanical limitations, beginning at the time of the insertion of the IPS-preprosthetic®. This is a completely novel approach to implant-borne prosthodontic restoration in difficult clinical situations, where much longer periods of protection were required before biomechanical loading could start. While the primary stability of dental implants is important in conventional implant dentistry, secondary stability must also be achieved in order for there to be reliable functioning [19]. However, it typically takes at least 12 weeks to achieve secondary stability in the compromised maxilla in most patients, especially in difficult clinical situations [20]. Furthermore, conventional implant dentistry protocols are almost always multistep surgical interventions. Often, they consist of bone augmentation, dental implant insertion, uncovering of the implant shoulder, and free mucosal grafting in cases where there is a lack of keratinized gingiva around the implant shoulder. Combined, it can take up to 12 months before the prosthodontics are fully completed. This again differs strongly from the presented protocol using IPS-preprosthetic®.

Only in one patient, in the case of an 18-year-old female patient, the combined IPS-preprosthetic® together with conventional dental implants was the initial treatment protocol for implant-borne prosthodontic restoration. In all other cases, IPS-preprosthetic® was used as a bailout strategy after a previously failed treatment protocol, making treatment easier.

**Fig. 7:**
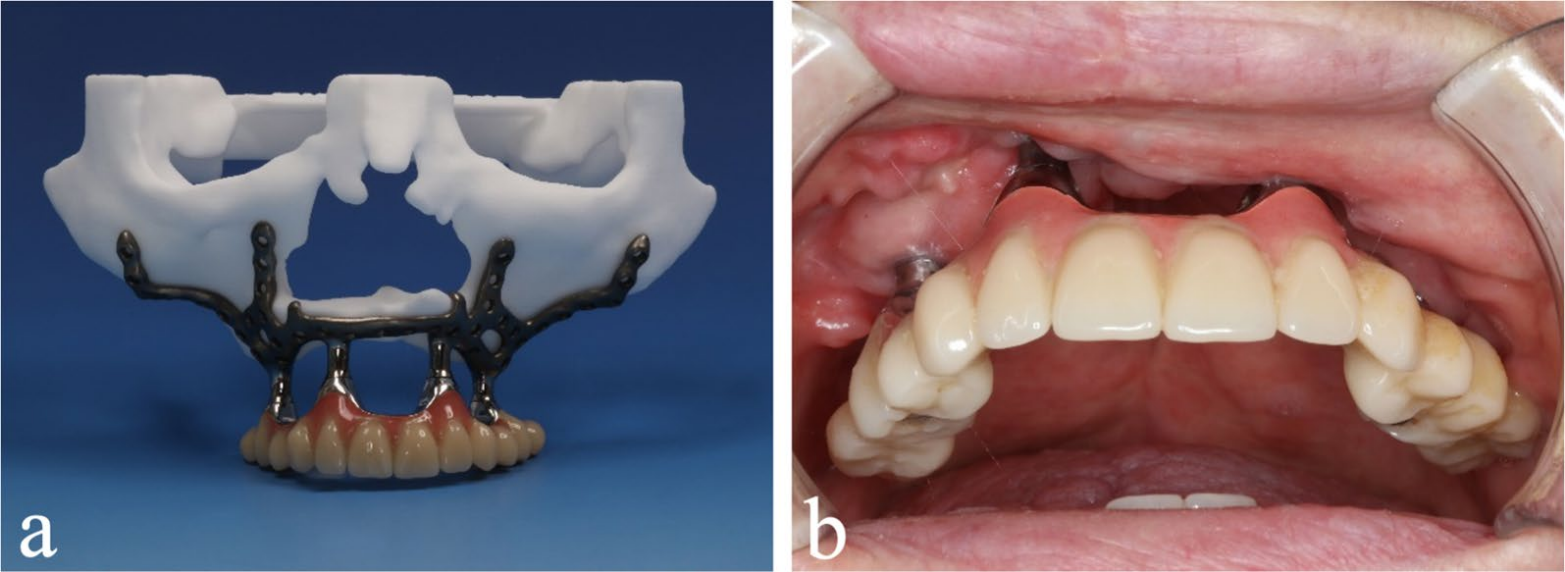
Temporary superstructure. The screw-retained temporary superstructure on the IPS-preprosthetic® mounted on a stereolithography model (**a**) and a clinical example (**b**)

In two cases, an irregular alveolar crest in the maxilla required trimming to improve an adequately designed interface to the underside of the IPS-preprosthetic® framework. Importantly, the cutting guides are simultaneously planned and manufactured from an autoclavable resin (polyamide) using 3D-printing technology. While this guide could also be screw-retained, given its excellent fit, including flanges, it could easily be combined with piezosurgery in order to precisely perform the resection. The advantage of the patient-specific implant is that this technically achieved form is already implemented in the final design of the IPS-preprosthetic®. This is yet another advantage of the implementation of modern 3D-technology in demanding cases of implant dentistry. Notably, since the planning of the final post position and vectors are defined once, no further abutment corrections to the finalized IPS-preprosthetic® are needed. The first temporary denture, which is basically a suprastructure made of a metallic bar mounted with acrylic teeth, can also be used to double-check the correct manufacturing of the implant on the one-hand side and a tension-free fixation on the other. In fact, this device is screw-retained and already mounted at the time of transoral insertion of the IPS-preprosthetic®; thus, during the multivector screw fixation, the post positions are saved (Fig. 7). For the final denture we prefer various types of removable partial or complete replacements for teeth, such as bar-clip retention or telescopic crowns (German crowns), since oral hygiene can be maintained significantly better hereby. In almost all cases, the soft tissues in the transition zone of the posts were not original keratinized gingiva. The additional soft tissue coverage seems to be the relevant factor. We see chronic mucositis in all cases, but so far there have not been any problems such as abscess or implant failure. We assume that this is mainly due to the use of coverage by a pedicled Bichat fat pad and the pre-described multivector distant fixation.

## Conclusions

Among all demanding cases of implant-borne dental rehabilitation, those following a failure of previously applied conventional methods, as well as those with significantly compromised soft and hard tissues, or a severe class III relationship, are the most challenging cases to treat. In addition to invasive protocols, which often take up to 1 year to achieve the final prosthodontic restoration, our concept based on a CAD/CAM protocol using an IPS-preprosthetic® implant offers a reliable and much quicker alternative strategy, allowing even for full primary loading.

However, as the presented technique is a novel approach for treatment in CLP patients, the number of patients that have received this implant is still limited. In order to determine any potential complications as well as assess the long-term efficacy of this novel approach, future studies using larger cohorts are needed. Nevertheless, our encouraging initial findings support the wider use of this novel technique.

## Data Availability

Data available on request from the authors.
